# Risk factor analysis and nomogram construction of postoperative complications for patients with locally advanced gastric cancer who received neoadjuvant immunotherapy and chemotherapy

**DOI:** 10.3389/fmed.2024.1405704

**Published:** 2024-07-26

**Authors:** Hao Cui, Sijin Zhang, Linde Sun, Zhen Yuan, Qixuan Xu, Jingwang Gao, Lin Chen, Jianxin Cui, Bo Wei

**Affiliations:** ^1^School of Medicine, Nankai University, Tianjin, China; ^2^Department of General Surgery, The First Medical Center, Chinese PLA General Hospital, Beijing, China; ^3^Department of Gastrointestinal Surgery, Peking University International Hospital, Beijing, China

**Keywords:** gastric cancer, neoadjuvant therapy, immunotherapy, nomogram, postoperative complication

## Abstract

**Introduction:**

The combination of neoadjuvant immunotherapy and chemotherapy (NICT) has become a common treatment regimen for locally advanced gastric cancer (LAGC). However, the safety and efficacy of radical gastrectomy following NICT (NICT-G) remain controversial. This study aimed to analyze the risk factors influencing postoperative complications (POCs) after NICT-G. Additionally, it aimed to construct a nomogram to provide a clinical reference for predicting POCs.

**Methods:**

This study included 177 patients who received NICT-G at the Chinese PLA General Hospital First Medical Center from January 2020 to January 2024. Univariable and multivariable logistic regression models were used to evaluate the risk factors influencing POCs, and a nomogram model was constructed. To evaluate the discrimination and accuracy of the nomogram model, the area under the receiver operating characteristic curve (AUC) and the calibration curve were measured.

**Results:**

In 177 patients who received NICT-G, the pathological complete response and major pathological response rates were 15.8% and 45.2%, respectively, whereas the rates of the overall and severe treatment-related adverse events were 71.8% and 15.8%, respectively. In addition, 43 (24.3%) patients developed overall POCs (Clavien–Dindo classification ≥ II). Univariable and multivariable logistic analyses showed that age ≥70 years, greater estimated blood loss, platelet/lymphocyte ratio (PLR) ≤196, neutrophil/lymphocyte ratio (NLR) >1.33, non-R0 resection, and body mass index (BMI) < 18.5 kg/m^2^ were independent risk factors for overall POCs (*p* < 0.05). The nomogram model developed using the abovementioned variables showed that the AUC (95% confidence interval [CI]) was 0.808 (95% CI): 0.731–0.885 in predicting the POC risk. The calibration curves showed that the prediction curve of the nomogram was a good fit for the actual POCs (Hosmer–Lemeshow test: χ^2^ = 5.76, *P* = 0.451).

**Conclusion:**

The independent risk factors for overall POCs in the NICT-G were age ≥ 70 years, greater estimated blood loss, PLR ≤ 196, NLR > 1.33, non-R0 resection, and BMI < 18.5 kg/m^2^. The nomogram model developed based on the abovementioned indicators showed better accuracy in predicting the POC risk.

## 1 Introduction

According to the statistics from GLOBOCAN 2020 (1), gastric cancer (GC) is the fifth most common cancer worldwide, and it poses a high risk of tumor-related deaths. China has a high incidence of GC, with locally advanced gastric cancer (LAGC) representing a significant portion GC cases in China (2). In addition to radical gastrectomy, performing perioperative treatments such as chemotherapy, immunotherapy, and targeted therapy is equally essential. These approaches achieve better therapeutic effects and improve the long-term prognosis for patients with LAGC.

One of the most crucial perioperative treatments for LAGC is neoadjuvant therapy. The concept of ypTNM stages, aimed at facilitating the broad application and precise evaluation of tumor regression after neoadjuvant therapy in LAGC, was first proposed in the GC staging criteria of the American Joint Committee on Cancer (AJCC), 8th edition. Recently, the combination of neoadjuvant immunotherapy and chemotherapy (NICT) has become the preferred treatment regimen for LAGC due to its superior tumor regression, acceptable treatment tolerance, and perioperative safety compared to using neoadjuvant chemotherapy alone (3, 4). The KEYNOTE-585 study demonstrated that patients who received NICT [pembrolizumab plus fluorouracil + oxaliplatin + docetaxel (FLOT)] did not reach the threshold for statistical significance (*p* = 0.0178) in event-free survival (5); however, a higher proportion of pathological complete response (pCR) indicated a potential survival benefit for patients with LAGC (6).

Surgeons rely on perioperative safety as a key factor when performing radical gastrectomy following NICT (NICT-G). Previous studies have reported that NICT-G and gastrectomy following neoadjuvant chemotherapy have comparable postoperative complications (POCs) and recovery (7, 8). However, based on our clinical experience, some potential risks caused by NICT-G may increase surgical difficulty and even lead to POCs, including a decreased immune system and physical status during NICT, increased tissue fragility, and disruptions of anatomical gaps. Therefore, this explorative retrospective study aimed to present the therapeutic effects of NICT, analyze the risk factors affecting POCs following NICT-G, and construct a nomogram to provide a clinical reference for predicting POCs following NICT-G.

## 2 Materials and methods

### 2.1 Patients

A total of 177 patients who received NICT-G in the Chinese PLA General Hospital First Medical Center from January 2020 to January 2024 were included in this study. The inclusion criteria were as follows: (1) patients in the cT_2_N _+_ M_0_ or T_3 − 4*b*_N_any_M_0_ (clinical TNM stages II–Iva) clinical stage, according to the AJCC 8th edition GC staging criteria; (2) those with pathologically confirmed gastric adenocarcinoma; and (3) those who received NICT simultaneously. The exclusion criteria were as follows: (1) patients who received targeted therapy, radiotherapy, or other antitumor treatments rather than NICT before surgery; (2) those with residual GC or a history of gastric surgery before NICT; (3) those with other gastric tumors, such as mesenchymal tumor and lymphoma; and (4) those lacking integrated clinical and pathological data. We collected data on sex, age, body mass index (BMI), age-adjusted Charlson Comorbidity Index (aCCI) score, the American Society of Anesthesiologists (ASA) grade, the Nutritional Risk Screening-2002 (NRS-2002) score, history of abdominal surgery, cTNM stages, neoadjuvant pathological tumor (ypT) stage, neoadjuvant pathological node (ypN) stages, ypTNM stages, tumor differentiation, tumor diameter, signet ring cell carcinoma, and vascular and nerve invasion as baseline characteristics (Tables 1, 2). The ethics committee of the Chinese PLA General Hospital First Medical Center approved this study (approval No. S2023-190-01), and all patients provided informed consent before perioperative treatment.

**Table 1:** Baseline characteristics of the NICT-G group.

**Baseline characteristics**	**NICT-G group (*n =* 177)**
**Sex**, ***n*** **(%)**	
Male	135 (76.3)
Female	42 (23.7)
Age, *mean ± SD* (years old)	60.92 ± 10.21
BMI, *mean ± SD* (kg/m^2^)	23.64 ± 3.30
**NRS-2002 score**, ***n*** **(%)**	
≥3	87 (49.2)
< 3	90 (50.8)
**ASA grade**, ***n*** **(%)**	
II	166 (93.8)
III	11 (6.2)
**History of abdominal surgery**, ***n*** **(%)**	
No	147 (83.1)
Yes	30 (16.9)
**aCCI grade**, ***n*** **(%)**	
< 5	114 (64.4)
≥5	63 (35.6)
**cTNM stage**, ***n*** **(%)**	
II	34 (19.2)
III	136 (76.8)
IVa	7 (4.0)

NICT-G, gastrectomy following neoadjuvant immunotherapy and chemotherapy; ASA, American Society of Anesthesiologists; BMI, body mass index; aCCI, age-adjusted Charlson Comorbidity Index; NRS-2002, Nutritional Risk Screening-2002; Mean ± SD, mean ± standard deviation.

**Table 2:** Pathological characteristics of the NICT-G group.

**Pathological characteristics**	**NICT-G group (*n =* 177)**
**ypT**, ***n*** **(%)**	
0	29 (16.4)
1	40 (22.6)
2	16 (9.0)
3	80 (45.2)
4	12 (6.8)
**ypN**, ***n*** **(%)**	
0	90 (50.8)
1	26 (14.7)
2	25 (14.1)
3	36 (20.3)
**ypTNM stage**, ***n*** **(%)**	
T_0_N_0_M_0_	28 (15.8)
I	50 (28.2)
II	36 (20.3)
III	63 (35.6)
**Tumor differentiation**, ***n*** **(%)**	
Well/Moderate	98 (55.4)
Poor/Undifferentiated	79 (44.6)
Tumor diameter, M (IQR)	3.0 (2.0–4.5)
**Signet-ring cell carcinoma**, ***n*** **(%)**	
No	136 (76.8)
Yes	41 (23.2)
**Vascular invasion**, ***n*** **(%)**	
No	136 (76.8)
Yes	41 (23.2)
**Nerve invasion**, ***n*** **(%)**	
No	125 (70.6)
Yes	52 (29.4)
**TRG grade**, ***n*** **(%)**	
Ia	28 (15.8)
Ib	52 (29.4)
II	60 (33.9)
III	37 (20.9)
pCR, *n* (%)	28 (15.8)
Major pathological response, *n* (%)	80 (45.2)

NICT-G, gastrectomy following neoadjuvant immunotherapy and chemotherapy; pCR, Pathological complete response; MPR, Major pathological response; TRG, Tumor regression grade; ypTNM: neoadjuvant pathological TNM stage.

### 2.2 Neoadjuvant therapy and therapeutic effect evaluation

All patients in this study accepted simultaneous chemotherapy and immunotherapy preoperatively. The following were the explicit neoadjuvant regimens: (1) SOX (S-1 combined with oxaliplatin), (2) XELOX (capecitabine combined with oxaliplatin) and SAP (S-1 combined with nab-paclitaxel), (3) FOLFOX (oxaliplatin combined with fluorouracil), and (4) FLOT (fluorouracil + oxaliplatin + docetaxel). Neoadjuvant immunotherapy regimens were totally programmed as death-1 inhibitors, which included nivolumab, pembrolizumab, toripalimab, camrelizumab, and sintilimab. During NICT, we used the Common Terminology Criteria for Adverse Events criteria (version 5.0) to define the category and severity of treatment-related adverse events (TRAEs) (9).

The therapeutic effects of NICT were evaluated using abdominal enhanced computed tomography (CT) and postoperative pathological results. Following the Response Evaluation Criteria in Solid Tumors criteria (version 1.1), abdominal enhanced CT scans were performed every 6 weeks preoperatively to evaluate the radiologic response. The Response Evaluation Criteria in Solid Tumors criteria (version 1.1) were divided into complete response (CR), partial response (PR), stable disease (SD), and progressive disease (PD) (10). The pathological TNM stages after neoadjuvant therapy were defined as ypTNM stages according to the 8th edition of the AJCC Cancer Staging Manual, which was especially used for tumor staging after neoadjuvant therapy. The ypTNM stages divided patients with non-metastatic LAGC after receiving neoadjuvant therapy into three stages: stage I, stage II, and stage III. The ypT and ypN stages were determined by the remaining tumor cells located in the deepest layer of the gastric wall and metastasis in the regional lymph nodes. The neoadjuvant pathological T_0_ (ypT_0_) and neoadjuvant pathological N_0_ (ypN_0_) stages indicated that no residual tumor existed in the gastric wall and regional lymph nodes, respectively, after neoadjuvant therapy. For evaluating the tumor regression grade (TRG) from pathological results, Becker's standard was used as follows: (1) TRG1a, no residual tumor cells; (2) TRG1b, < 10% residual tumor cells; (3) TRG2, 10%−50% residual tumor cells; and (4) TRG3, >50% residual tumor cells (11). pCR and major pathological response (MPR) were defined as TRG1a (ypT_0_N_0_M_0_) and TRG1a/1b, respectively.

### 2.3 Perioperative indicators

Previous studies have suggested that preoperative laboratory indices hold value in POC prediction (12, 13). Therefore, we collected data on the hemoglobin level, leukocyte, neutrophil, lymphocyte, and platelet counts, and the serum albumin level in the peripheral blood. Furthermore, we calculated combined laboratory indicators, including the neutrophil/lymphocyte ratio (NLR, neutrophil count/lymphocyte count), the platelet/lymphocyte ratio (PLR, platelet count/lymphocyte count), Onodera's prognostic nutritional index (PNI) score (10 × albumin [g/dL] + 0.005 × lymphocyte count/mm^3^) (14), and the systemic immune-inflammation (SII) index score (platelet count × neutrophil count/lymphocyte count) (15).

### 2.4 Surgery and postoperative recovery

Radical gastrectomy plus D2 lymphadenectomy was performed 4–6 weeks following the completion of NICT. The surgical details were consistent with the Japanese GC Treatment Guidelines (6th edition) (16). Operation time and estimated blood loss were recorded during the NICT-G, and data on the retrieved lymph nodes, first flatus days, postoperative hospitalized days, and 30-day POCs were collected from the postoperative medical records and follow-up visits. The Clavien–Dindo classification was used to define POC severity (17). Owing to the limitations of a retrospective study, we defined POCs ≥Clavien–Dindo Grade II as overall POCs and POCs ≥Clavien–Dindo Grade IIIa as severe POCs. Postoperative treatment was initiated 4 weeks after the operation.

### 2.5 Variables for risk factors

We conducted a binary logistic regression analysis to explore the risk factors for the overall POCs after the NICT-G. The cut-off values of the BMI, PNI, NLR, PLR, and SII index were selected using the Youden Index from the receiver operating characteristic (ROC) curve and clinical preference. The following clinical and operative variables were investigated as possible risk factors for POCs after the NICT-G: sex (male or female), age (< 70 years or ≥70 years), BMI (< 18.5 kg/m^2^, 18.5 to < 24.0 kg/m^2^, 24.0 to < 28.0 kg/m^2^, or ≥28.0 kg/m^2^), tumor resection (proximal, distal, or total), ASA grade (II or III), severe TRAEs (yes or no), aCCI score (< 5 or ≥5), NRS-2002 score (< 3 or ≥3), surgical approach (open, laparoscopic, or robotic), R0 resection (yes or no), tumor diameter (< 2 cm or ≥2 cm), operation time (< 250 min or ≥250 min), PNI score (< 45 or ≥45), SII index (≤ 260 or >260), NLR (≤ 1.33 or >1.33), and PLR (≤ 196 or >196).

### 2.6 Statistical analysis

All statistical analyses were performed using the Statistical Package for the Social Sciences version 26.0 (IBM SPSS Statistics, Chicago, IL, USA) and R version 4.2.2 (R Project, the Institute of Statistics and Mathematics, Vienna, Austria). Continuous data with normal distribution were expressed as *means* ± *standard deviations*, whereas categorical variables were presented as numbers (percentages). Continuous data with skewed distribution were expressed as medians (interquartile ranges). A binary logistic regression analysis was conducted for variable analysis, and factors with a *p-*value of < 0.15 were included in the multivariable logistic analysis. Independent risk factors were used to construct a nomogram prediction model using R packages. We used the 1,000 bootstrap replications method for drawing the calibration curve; subsequently, the Hosmer–Lemeshow goodness-of-fit test was conducted to evaluate the goodness of fit. The predictive performance of the risk model was shown using the ROC curve and quantified using the area under the ROC curve [area under the receiver operating characteristic curve (AUC)]. A *p-*value of < 0.05 was considered statistically significant.

## 3 Results

### 3.1 Clinicopathological characteristics

Overall, 177 patients with LAGC who underwent NICT-G were enrolled in the final analysis. The clinicopathological characteristics of the enrolled patients are presented in Tables 1, 2. Most of the enrolled patients were male individuals (76.3%), with an average age of 60.92 ± 10.21 years and a BMI of 23.64 ± 3.30 kg/m^2^. Of the enrolled patients, 34 (19.2%) were classified as having cTNM stage II, 136 (76.8%) as having cTNM stage III, and 7 (4.0%) as having cTNM stage Iva.

Regarding pathological characteristics, the patients were classified into ypTNM stages as follows: 28 (15.8%) were at stage T_0_N_0_M_0_, 50 (28.2%) were at stage I, 36 (20.3%) were at stage II, and 63 (35.6%) were at stage III. The median tumor diameters were 3.0 (2.0–4.5) cm. Furthermore, 41 (23.2%) of the enrolled patients had vascular invasion and 52 (29.4%) had nerve invasion. According to Becker's standard, which evaluated the TRG, 28 (15.8%) patients acquired TRG1a with pCR, while 80 (45.2%) patients acquired TRG1a/1b with MPR.

### 3.2 Treatment regimens and TRAEs

The percentage of patients who received ≥4 cycles of NICT was 74.6% (132/177 patients). Most patients (82.5%) accepted domestic neoadjuvant immunotherapy regimens (e.g., sintilimab, tislelizumab, and camrelizumab). The proportions of complete response (CR), partial response (PR), stable disease (SD), and progressive disease (PD), which reflected the radiological response, were 8.5%, 57.6%, 29.9%, and 4.0%, respectively. The objective response and disease control rates were 66.1% and 96.0%, respectively (Table 3).

**Table 3:** Regimens, cycles, and radiological responses of the NICT group.

**Variables**	**NICT-G group (*n =* 177)**
**Neoadjuvant chemotherapy**, ***n*** **(%)**	
SOX	114 (64.4)
XELOX	44 (24.9)
SAP	10 (5.6)
Others	9 (5.1)
**Neoadjuvant immunotherapy**, ***n*** **(%)**	
Domestic drugs	146 (82.5)
Imported drugs	31 (17.5)
**NICT cycle**, ***n*** **(%)**	
< 4	45 (25.4)
≥4	132 (74.6)
**Radiological response**, ***n*** **(%)**	
CR	15 (8.5)
PR	102 (57.6)
SD	53 (29.9)
PD	7 (4.0)
Objective response rate, *n* (%)	117 (66.1)
Disease control rate, *n* (%)	170 (96.0)

NICT-G, gastrectomy following neoadjuvant immunotherapy and chemotherapy; CR, Complete response; PR, Partial response; SD, Stable disease; PD, Progressive disease; ORR, Objective response rate; DCR, Disease control rate; SOX, S-1 combined with oxaliplatin; XELOX, Capecitabine combined with oxaliplatin; SAP, S-1 combined with nab-paclitaxel.

The TRAEs experienced by the patients during NICT are presented in Table 4. Leukopenia, nausea and vomiting, thrombocytopenia, and neutropenia were the top four most common TRAEs during the NICT. The rates of the overall and severe TRAEs were 71.8% and 15.8%, respectively. No patients died during the NICT.

**Table 4:** Treatment-related adverse events during NICT.

**Adverse events**	**Grade I**	**Grade II**	**Grade III**	**Grade IV**
Leukopenia	14	15	4	0
Nausea and vomiting	14	14	3	0
Maculopapular rash	0	3	3	0
Alopecia	0	1	0	0
Hematemesis	0	0	1	0
Diarrhea	2	1	0	0
Thrombocytopenia	9	19	6	1
Neutropenia	11	7	7	0
Hand-foot syndrome	0	2	0	0
Anemia	1	9	1	0
Hypothyroidism	0	2	0	0
Dysphasia	0	1	0	0
Elevated ALT/AST	6	4	2	0
Overall TRAEs rates, *n* (%)	127 (71.8)			
Severe TRAEs rates, *n* (%)	28 (15.8)			

NICT-G, gastrectomy following neoadjuvant immunotherapy and chemotherapy; TRAEs, Treatment-related adverse events; POCs, Postoperative complications.

### 3.3 Surgical safety, postoperative recovery, and complications

The perioperative treatment and recovery of the patients who received NICT-G are shown in Table 5. Patients in the NICT-G group required an average of 235.95 ± 57.14 min of operation time, and the estimated blood loss was 100.0 (50.0–100.0) mL. Most patients (94.9%) accepted minimally invasive gastrectomy following NICT, and the R0 resection rate of all the enrolled patients was 93.8%. The first flatus and the postoperative hospitalized days were 4.0 (3.0–4.0) and 8.0 (7.0–10.0) days, respectively.

**Table 5:** Perioperative variables and postoperative recovery in the NICT-G group.

**Variables**	**NICT-G group (*n =* 177)**
Operation time, *mean ± SD* (min)	235.95 ± 57.14
Estimated blood loss. ml, M (IQR)	100.0 (50.0–100.0)
**R0 resection**, ***n*** **(%)**	
R0	166 (93.8)
R1	11 (6.2)
**Surgical approach*****, n*** **(%)**	
Open gastrectomy	9 (5.1)
Laparoscopic gastrectomy	136 (76.8)
Robotic gastrectomy	32 (18.1)
**Tumor resection**, ***n*** **(%)**	
Proximal gastrectomy	47 (26.5)
Distal gastrectomy	51 (28.8)
Total gastrectomy	79 (44.6)
**Combined organ resection**, ***n*** **(%)**	
No	173 (97.7)
Yes	4 (2.3)
Retrieved lymph nodes, *n*, M (IQR)	27.0 (21.0–37.0)
First flatus days, d, M (IQR)	4.0 (3.0–4.0)
Postoperative hospitalized days, d, M (IQR)	8.0 (7.0–10.0)

NICT-G, gastrectomy following neoadjuvant immunotherapy and chemotherapy; M (IQR), Median (interquartile range); Mean ± SD, Mean ± standard deviation.

Moreover, 43 (24.3%) patients developed POCs (Clavien–Dindo classification ≥ II) (Table 6). Hypoproteinemia (11/43), anemia (9/43), anastomotic leakage (4/43), and anastomotic hemorrhage (4/43) were the four most common complications. The prevalence of severe complication (Clavien–Dindo classification ≥ IIIa) and postoperative mortality were 7.3% and 1.1%, respectively.

**Table 6:** Clavien–Dindo classification of postoperative complications in the NICT-G group.

**Complications**	**Grade II**	**Grade IIIa**	**Grade IV**	**Grade V**
Atrial fibrillation	3	0	0	0
Anastomotic leakage	2	2	0	0
Anastomotic hemorrhage	2	1	1	0
Hypoproteinemia	11	0	0	0
Pancreatic leakage	2	1	0	0
Anemia	9	0	0	0
Elevated transaminase	2	0	0	0
Thrombocytopenia	1	0	0	0
Electrolyte disturbance	1	0	0	0
Pneumothorax	0	1	0	0
Pleural effusion	0	2	0	0
Abdominal abscess	0	1	0	0
Pleural infection	0	0	1	0
Herniation of intestine	0	0	1	0
Pulmonary embolism	0	0	0	1
Cardiac shock	0	0	0	1
Overall postoperative complications, *n* (%)	43 (24.3)			
Severe postoperative complications, *n* (%)	13 (7.3)			
Postoperative 30-day mortality, *n* (%)	2 (1.1)			

NICT-G, gastrectomy following neoadjuvant immunotherapy and chemotherapy.

### 3.4 Risk factors for POCs in the NICT-G group

**Figure 1:**
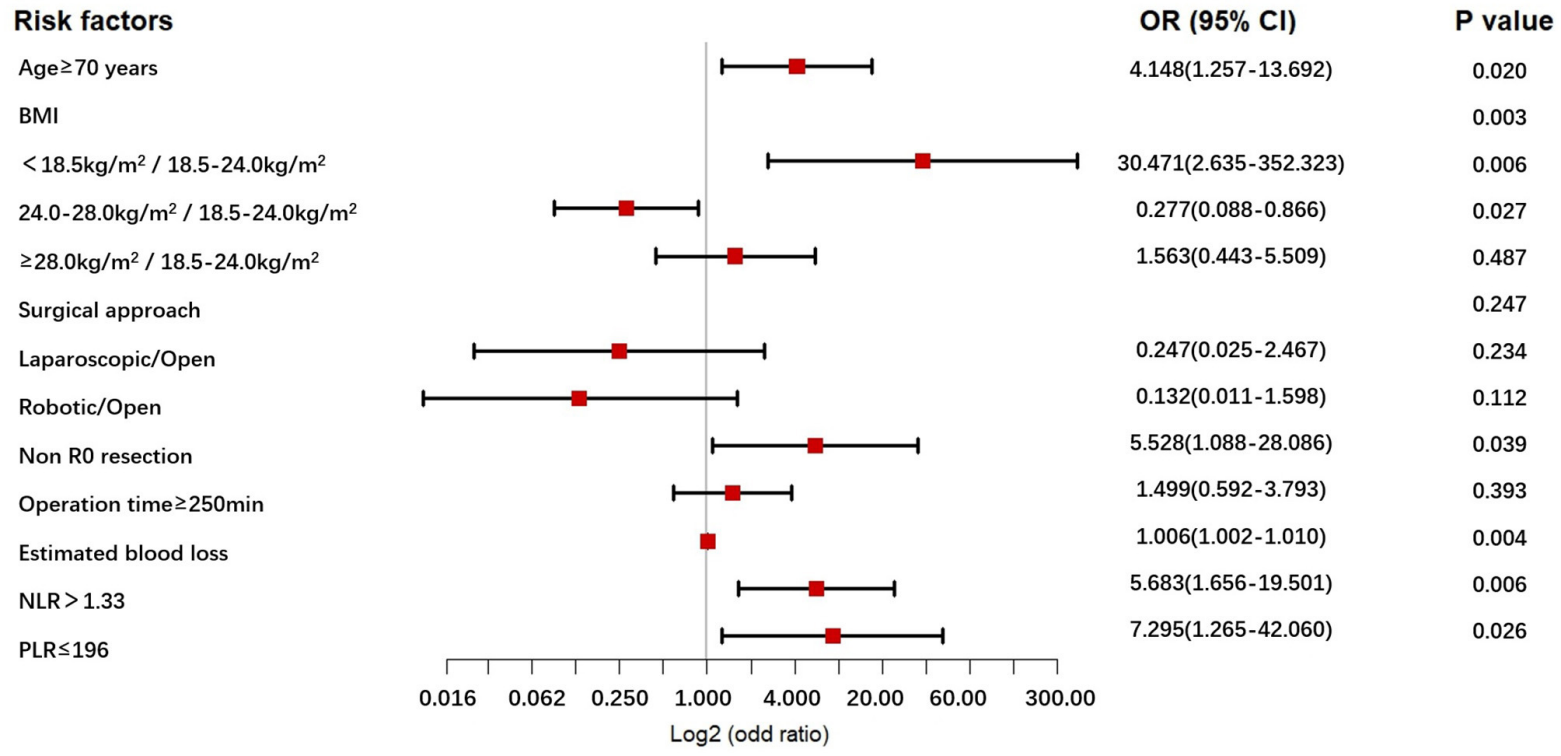
The forest plot for multivariable analysis for POCs in the NICT-G patients. POCs, postoperative complications; NICT-G, gastrectomy following neoadjuvant immunotherapy and chemotherapy.

The univariable - and multivariable logistic regression results used to explore the risk factors for POCs following NICT-G are shown in Table 7 and Figure 1. We placed indicators obtained by the univariable logistic regression with a *p*-value of < 0.15 into the multivariable analysis and observed that age ≥70 years (odds ratio [OR] [95% confidence interval (CI)]: 4.148 [1.257–13.692], *P* = 0.020), greater estimated blood loss (OR [95% CI]: 1.006 [1.002–1.010], *P* = 0.004), PLR ≤ 196 (OR [95% CI]: 7.295 [1.265–42.060], *P* = 0.026), NLR > 1.33 (OR [95% CI]: 5.683 [1.656–19.501], *P* = 0.006), non-R0 resection (OR [95%CI]: 5.528 [1.088–28.086], *P* = 0.039), and BMI < 18.5 kg/m^2^ (OR [95% CI]: 30.471 [2.635–352.323], *P* = 0.006) were significant independent risk factors for overall POCs.

**Table 7:** The characteristics of and univariable analysis of LAGC patients with and without POCs after NICT-G.

**Variables**	**Total patients (*n =* 177)**	**Patients without POCs (*n =* 134)**	**Patients with POCs (*n =* 43)**	**OR**	**95%CI**	***p*-value**
**Sex**						0.460
Male	135	104	31	1.000		
Female	42	30	12	1.342	0.615–2.929	
**Age (years)**						0.100
< 70	143	112	31	1.000		
≥70	34	23	12	1.971	0.878–4.421	
**BMI (kg/m** ^2^ **)**						0.008
18.5 to < 24.0	96	72	24	1.000		
< 18.5	7	1	6	18.783	2.148–164.245	0.008
24.0 to < 28.0	53	47	6	0.466	0.185–1.173	0.105
≥28.0	21	14	7	1.565	0.564–4.347	0.390
**aCCI index**						0.535
< 5	114	88	26	1.000		
≥5	63	46	17	1.251	0.616–2.538	
**Tumor resection**						0.725
Proximal	47	35	12	1.000		
Distal	51	37	14	1.104	0.449–2.712	0.830
Total	79	62	17	0.800	0.343–1.866	0.605
**ASA grade**						0.812
II	166	126	40	1.000		
III	11	8	3	1.181	0.299–4.666	
**Severe TRAEs**						0.566
No	149	114	35	1.000		
Yes	28	20	8	1.303	0.528–3.215	
**Surgical approach**						0.025
Open	9	3	6	1.000		
Laparoscopic	136	105	31	0.148	0.035–0.625	0.009
Robotic	32	26	6	0.115	0.022–0.598	0.010
**NRS-2002 score**						0.768
< 3	90	66	24	1.000		
≥3	87	68	19	0.768	0.385–1.533	
**R0 resection**						0.103
Yes	167	129	38	1.000		
No	10	5	5	2.807	0.812–9.708	
**Tumor diameter (cm)**						0.725
< 2 cm	26	19	7	1.000		
≥2 cm	151	115	36	1.180	0.471–2.957	
**Operation time**						0.107
< 250 min	97	74	23	1.000		
≥250 min	80	60	20	1.779	0.884–3.579	
Estimated blood loss	100.0 (50.0–100.0)	100.0 (50.0–100.0)	100.0 (50.0–300.0)	1.005	1.002–1.008	0.000
**PNI score**						0.576
< 45	44	22	22	1.000		
≥45	133	112	21	0.822	0.413–1.635	
**NLR**						0.060
≤ 1.33	49	42	7	1.000		
>1.33	128	92	36	2.348	0.966–5.706	
**PLR**						0.111
>196	21	19	2	1.000		
≤ 196	156	115	41	3.387	0.756–15.179	
**SII index**						0.198
>260	96	69	27	1.000		
≤ 260	81	65	16	0.629	0.311–1.273	

NICT-G, gastrectomy following neoadjuvant immunotherapy and chemotherapy; BMI, Body mass index; aCCI, Age-adjusted Charlson Comorbidity Index; NRS-2002, Nutritional Risk Screening-2002; NLR, Neutrophil-lymphocyte ratio; PLR, Platelet-lymphocyte ratio; PNI, Onodera's prognostic nutritional index; SII, Systemic immune-inflammation; TRAEs, Treatment-related adverse events; POCs, Postoperative complications; OR, Odds ratio; ASA, American Society of Anesthesiologists.

### 3.5 Construction and validation of the nomogram for predicting POCs

**Figure 2:**
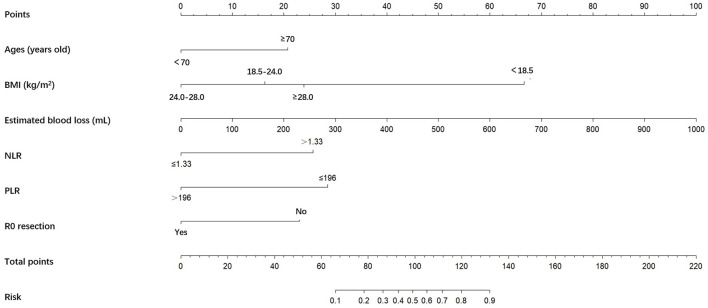
A nomogram predicting the risk of POCs for patients with NICT-G. The point for each factor was according to the condition of patients and odds ratio. The sum of the scores for each item is equal to the total points, then find the corresponding point on the total points axis and make a vertical line down to predict the risk of the POCs after NICT-G. POCs, postoperative complications; NICT-G, gastrectomy following neoadjuvant immunotherapy and chemotherapy.

**Figure 3:**
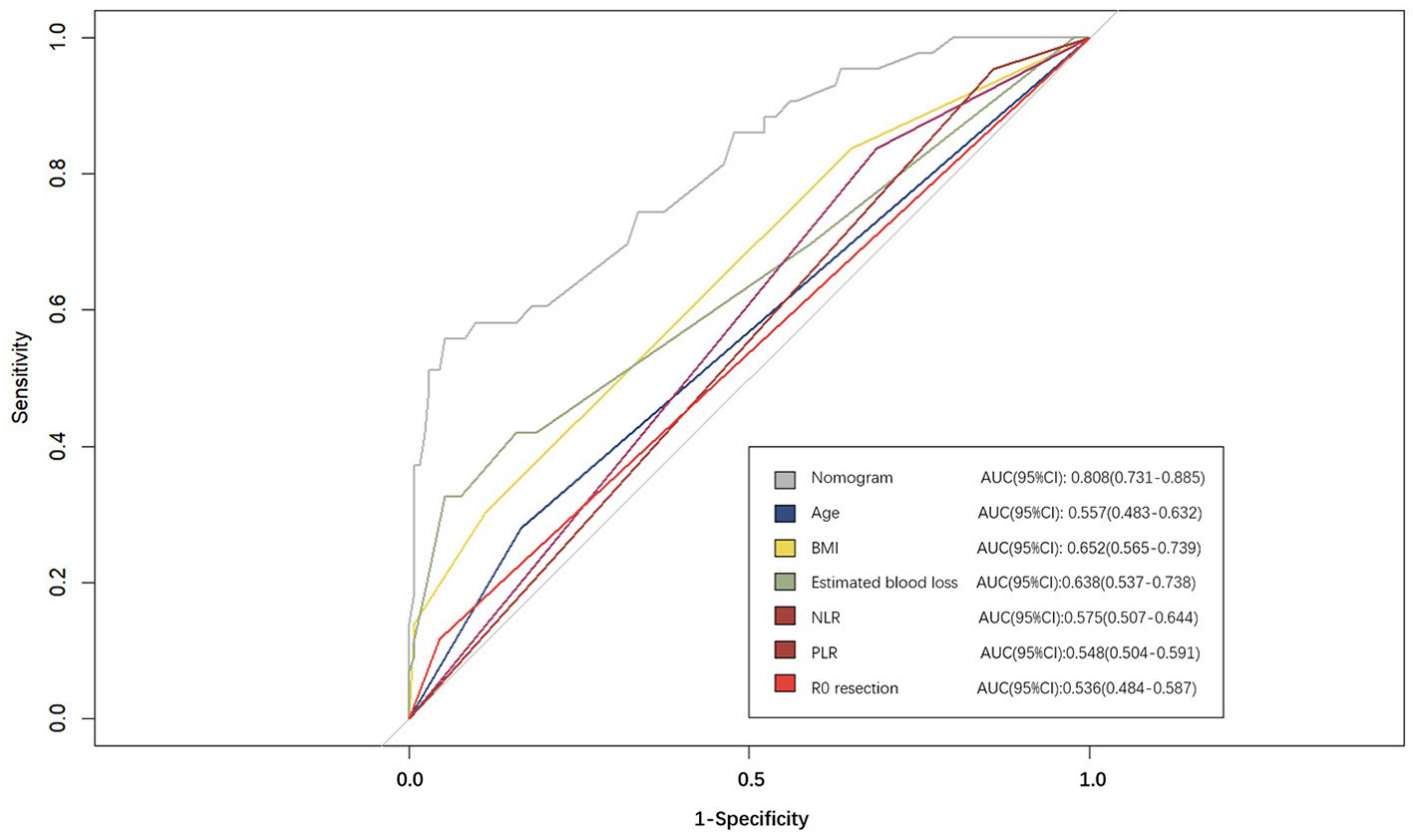
The receiver operating characteristic curves of nomogram and different risk factors.

**Figure 4:**
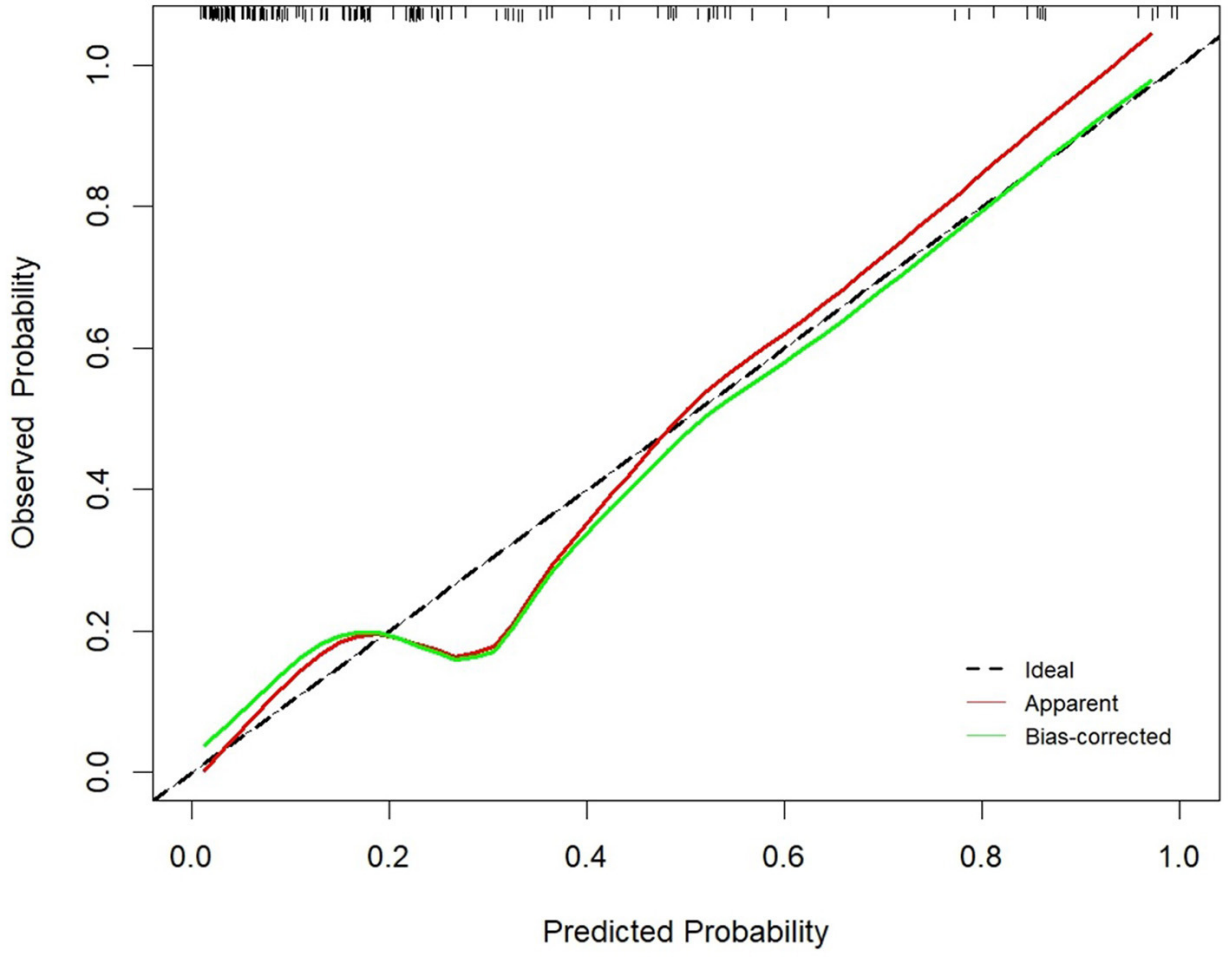
The calibration curve of the nomogram.

**Figure 5:**
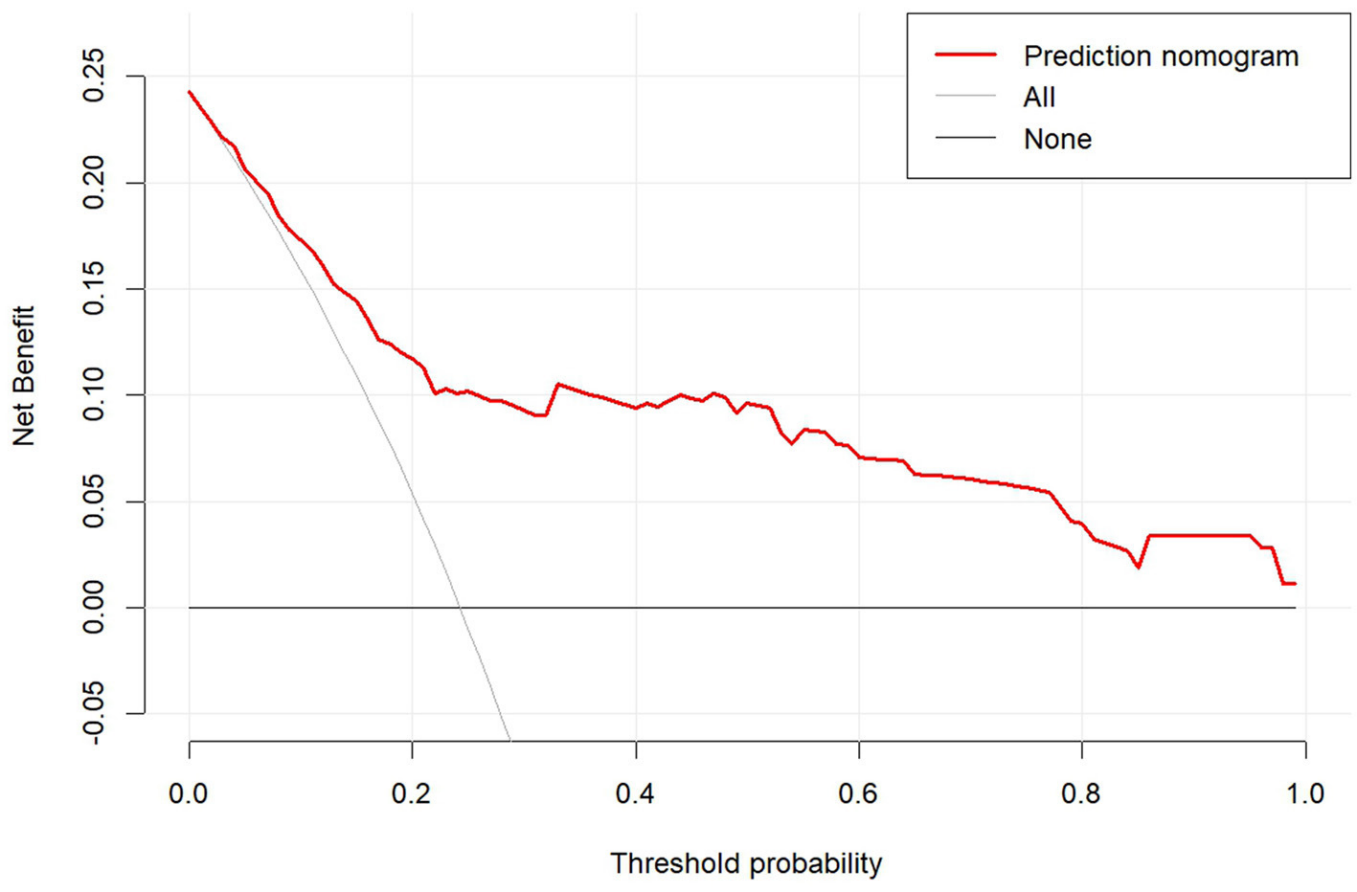
The decision curve analysis of the nomogram.

A nomogram was constructed based on the six independent predictive factors derived from the multivariable analysis (Figure 2). The corresponding point for each factor was assigned based on the condition of the patients and the OR, which could be calculated by drawing a vertical line upward (e.g., a patient with NLR > 1.33 would receive 26 points). The sum of the points for each item equaled the total score. Then, the corresponding point on the total point axis was located, and a vertical line was drawn downward to predict the risk of POCs after NICT-G. The ROC curve showed that the AUC of the combination of six variables was 0.808 (95% CI): 0.731–0.885, which was superior to any single risk factor (Figure 3). The calibration plot based on the 1,000 bootstrap replications method showed good adaptability between the prediction and observation (Hosmer–Lemeshow test: χ^2^ = 5.76, *P* = 0.451) (Figure 4). The decision curve analysis demonstrated that our predictive model showed good benefits for different threshold probabilities between 10% and 100%, indicating favorable clinical utility (Figure 5).

## 4 Discussion

In the last 2 years, NICT for patients with LAGC has been gradually gaining recognition owing to its superior antitumor effects compared with neoadjuvant chemotherapy alone. However, it remains unclear whether NICT affects surgical safety and elevates the potential risk of POCs. In the study, we observed that NICT showed satisfactory tumor regression and acceptable TRAEs in 177 patients with LAGC. Meanwhile, six independent risk factors for predicting POCs were detected, and a risk prediction model for predicting POCs in patients receiving NICT-G was successfully developed, which could facilitate preoperative assessment to elevate perioperative safety.

Medication safety and antitumor efficacy are crucial for promoting the application of NICT. A recent meta-analysis of 1,074 patients with resectable gastric/gastroesophageal junction tumors demonstrated that patients receiving NICT had a 24% pCR rate and a 49% MPR rate, with a 28% incidence of severe TRAEs (18). A study by Yuan et al. involving 54 patients with LAGC treated with NICT (toripalimab combined with SOX/XELOX) showed that a higher proportion of patients achieved TRG0/1 and exhibited a manageable safety profile compared to those treated with chemotherapy alone (3). Better tumor regression may translate into survival benefit. Lin et al. reported that the proportions of ypT0, ypN0, and pCR rates in the NICT (camrelizumab + nab-paclitaxel + S-1) group were significantly higher. The patients in the NICT group had a longer average time to recurrence (18.9 vs. 13.1 months, *P* = 0.004) than those in the chemotherapy group (19). In our NICT-G cohort, 15.8% of the patients achieved pCR and 45.2% achieved MPR, indicating higher rates than those reported in previous studies involving patients with LAGC treated with neoadjuvant chemotherapy (3, 20). The acceptable incidence of the overall and severe TRAEs (71.8% and 15.8%, respectively) further supports the safety and efficacy of NICT among patients with LAGC.

Surgeons focus more on surgical safety and postoperative recovery following neoadjuvant therapy. Our findings showed that patients in the NICT-G group had an average operation time of 235.95 ± 57.14 min and an estimated blood loss of 100.0 (50.0–100.0) mL, which were consistent with our previous studies on upfront gastrectomy (21). The values from these studies were also acceptable in terms of the first flatus and postoperative hospitalized days in the NICT-G group. We attribute these positive results to the high proportion of minimally invasive gastrectomy (94.9%) and the use of enhanced recovery after surgery (ERAS) in our cohort. The use of high-definition magnified imaging and precise surgical procedures during laparoscopy or the use of surgical robots may help counteract the negative effects of tissue brittleness, increased exudation, and blurred anatomical gaps caused by NICT, while the application of ERAS can promote the postoperative recovery of gastrointestinal function (22).

POCs are key indicators for evaluating short-term outcomes in patients with NICT-G. In this study, 43 (24.3%) patients developed POCs with a Clavien–Dindo classification of ≥II. Moreover, hypoproteinemia, anemia, anastomotic leakage, and anastomotic hemorrhage were the four most common POCs in the NICT-G group, suggesting that surgeons did not pay attention to the perioperative nutritional status, anastomotic safety, and exact hemostasis. We detected six risk factors through the multivariable analysis and developed a prediction model to assess the potential risk of POCs following NICT-G. Our nomogram showed good calibration (Hosmer–Lemeshow test: χ^2^ = 5.76, *P* = 0.451), excellent predictive ability (0.808 [95% CI: 0.731–0.885]), and good positive net benefit.

Previous studies have suggested that some laboratory indices, including NLR, PLR, PNI, and SII, play a certain role in the prediction of POCs (23, 24). A study conducted by Wang found that a higher NLR was an independent risk factor for infectious POCs following gastrectomy (25). Furthermore, Inaoka et al. reported that the low PLR group exhibited an increased incidence of POCs for clinical T_2 − 4_ GC (26). In our study, although the TRAEs of myelosuppression caused by NICT may have affected the changes in preoperative laboratory indicators, we noted that a lower preoperative PLR and a higher preoperative NLR were independent risk factors for POCs following NICT-G, indicating a good predictive value. This result was likely due to the ability of NLR and PLR to reflect systemic inflammatory responses, immune disorders, and potential microvascular thrombosis, which are strongly associated with POCs, particularly infectious POCs (27).

Older patients are prone to developing POCs owing to the high proportion of comorbidity and decreased physical status. A study by the Dutch Upper Gastrointestinal Cancer Audit group proved that age ≥ 70 years is an independent risk factor for predicting POCs following radical gastrectomy (28). Moreover, Yu et al. showed a close correlation between POCs and older age in patients who underwent gastrectomy following neoadjuvant chemotherapy (29). Although neoadjuvant therapy does not increase surgical complications following gastrectomy (30), the dual influence of NICT combined with surgery may increase the risk of POCs, especially for older patients. The results of our study showed that age ≥ 70 years was strongly associated with POCs following NICT-G, indicating that surgeons must emphasize the perioperative management of older patients. The requirements for the perioperative management of older patients are as follows: (1) a dynamic evaluation of the nutritional status with proper intervention, (2) effective treatment for TRAEs during NICT, (3) symptomatic treatment for comorbidities, and (4) a comprehensive preoperative assessment of the physical status by a multidisciplinary team (31).

According to the Chinese BMI classification, underweight is defined as < 18.5 kg/m^2^, normal weight as 18.5 to < 24 kg/m^2^, overweight as 24 to < 28 kg/m^2^, and obesity as ≥28 kg/m^2^ (32). A lower BMI score often reflects poor nutritional status, especially for patients who undergo NICT before surgery. Hirahara et al. found that a lower BMI score was associated with impaired nutritional status among patients and that the patients subsequently developed POCs (33). Kim's study indicated that a lower BMI score was an independent risk factor for pulmonary POCs after neoadjuvant therapy followed by surgery for non-small-cell lung cancer (34). Cohen et al. observed that neoadjuvant therapy-related nutritional deterioration increased the risk of POCs after cystectomy (35). Moreover, other previous studies have also demonstrated that patients with a lower BMI score have a higher risk of developing Petersen's hernia, experiencing more severe POCs, and having poorer prognosis after gastrectomy (36, 37). In our study, we found that patients with a lower BMI score of < 18.5 kg/m^2^ were prone to developing POCs such as hypoproteinemia and anemia, which indicated poor nutritional status during perioperative periods. Thus, based on mature minimally invasive technology and stable surgical safety, attention should be paid to perioperative nutritional assessment and symptomatic supportive treatment to reduce the risk of POCs in patients who undergo NICT-G.

This study had some limitations. First, selection bias may have existed in this single-institutional retrospective study. Second, POCs with a Clavien–Dindo classification of grade I were not included in the analysis owing to the limitations of a retrospective cohort (38). To systematically evaluate POCs following NICT-G, prospective studies should be conducted. Third, the effect of NICT on POCs was not included in this study. Future studies should focus on comparing the short-term outcomes between patients with LAGC receiving NICT and patients with LAGC not receiving NICT before gastrectomy. Finally, this study lacked external validation due to the limited sample size. Future studies should perform external validation, which could demonstrate the exact predictive value of the nomogram (39).

## 5 Conclusion

This study demonstrated the antitumor effects, medication safety, and perioperative recovery of patients with LAGC who underwent NICT-G in a large single-institutional cohort. Furthermore, we analyzed six independent risk factors, including age ≥ 70 years, greater estimated blood loss, PLR ≤ 196, NLR > 1.33, non-R0 resection, and BMI < 18.5 kg/m^2^, which could predict POCs following NICT-G. Meanwhile, we developed a new nomogram prediction model with good discrimination and calibration. This model could assist surgeons in individualizing perioperative management by predicting the potential risk of POC development after NICT-G.

## Data availability statement

The raw data supporting the conclusions of this article will be made available by the authors on reasonable request, without undue reservation.

## Ethics statement

This study was approved by the Ethics Committee of the Chinese PLA General Hospital (approval No. S2023-190-01). The studies were conducted in accordance with the local legislation and institutional requirements. The human samples used in this study were acquired from Electronic medical record system. Written informed consent for participation was not required from the participants or the participants' legal guardians/next of kin in accordance with the national legislation and institutional requirements.

## Author contributions

HC: Conceptualization, Data curation, Visualization, Writing – original draft, Writing – review & editing. SZ: Conceptualization, Data curation, Writing – original draft. LS: Conceptualization, Data curation, Writing – original draft, Writing – review & editing. ZY: Data curation, Writing – original draft, Writing – review & editing. QX: Data curation, Writing – review & editing. JG: Data curation, Writing – review & editing. LC: Funding acquisition, Supervision, Validation, Writing – review & editing. JC: Conceptualization, Funding acquisition, Supervision, Validation, Writing – review & editing. BW: Conceptualization, Data curation, Funding acquisition, Supervision, Validation, Writing – review & editing.
